# Retraction: Dissection of soybean populations according to selection signatures based on whole-genome sequences

**DOI:** 10.1093/gigascience/giab038

**Published:** 2021-05-11

**Authors:** Jae-Yoon Kim, Seongmun Jeong, Kyoung Hyoun Kim, Won-Jun Lim, Ho-Yeon Lee, Namhee Jeong, Jung-Kyung Moon, Namshin Kim

**Affiliations:** Genome Editing Research Center, Korea Research Institute of Bioscience and Biotechnology (KRIBB), Gwahak-ro 125, Yuseong-gu, Daejeon 34141, Republic of Korea; Department of Bioinformatics, KRIBB School of Bioscience, University of Science and Technology (UST), Gajeong-ro 217, Yuseong-gu, Daejeon 34141, Republic of Korea; Genome Editing Research Center, Korea Research Institute of Bioscience and Biotechnology (KRIBB), Gwahak-ro 125, Yuseong-gu, Daejeon 34141, Republic of Korea; Genome Editing Research Center, Korea Research Institute of Bioscience and Biotechnology (KRIBB), Gwahak-ro 125, Yuseong-gu, Daejeon 34141, Republic of Korea; Department of Bioinformatics, KRIBB School of Bioscience, University of Science and Technology (UST), Gajeong-ro 217, Yuseong-gu, Daejeon 34141, Republic of Korea; Genome Editing Research Center, Korea Research Institute of Bioscience and Biotechnology (KRIBB), Gwahak-ro 125, Yuseong-gu, Daejeon 34141, Republic of Korea; Department of Bioinformatics, KRIBB School of Bioscience, University of Science and Technology (UST), Gajeong-ro 217, Yuseong-gu, Daejeon 34141, Republic of Korea; Genome Editing Research Center, Korea Research Institute of Bioscience and Biotechnology (KRIBB), Gwahak-ro 125, Yuseong-gu, Daejeon 34141, Republic of Korea; Department of Bioinformatics, KRIBB School of Bioscience, University of Science and Technology (UST), Gajeong-ro 217, Yuseong-gu, Daejeon 34141, Republic of Korea; National Institute of Crop Science, Rural Development Administration, Nongsaengmyeong-ro 370, Deokjin-gu, Jeon-Ju 54874, Republic of Korea; National Institute of Crop Science, Rural Development Administration, Nongsaengmyeong-ro 370, Deokjin-gu, Jeon-Ju 54874, Republic of Korea; Genome Editing Research Center, Korea Research Institute of Bioscience and Biotechnology (KRIBB), Gwahak-ro 125, Yuseong-gu, Daejeon 34141, Republic of Korea; Department of Bioinformatics, KRIBB School of Bioscience, University of Science and Technology (UST), Gajeong-ro 217, Yuseong-gu, Daejeon 34141, Republic of Korea

In December 2019, *GigaScience* published the article “Dissection of soybean populations according to selection signatures based on whole-genome sequences,” by Jae-Yoon Kim and colleagues: https://doi.org/10.1093/gigascience/giz151. In 2020, the editors received credible information about attribution problems with the source of the data in the article and subsequently alerted the readers with a statement of concern: https://doi.org/10.1093/gigascience/giaa040. A full institutional investigation has now been completed, which has concluded that research misconduct was carried out. As a result, this article is being retracted by the editors and authors.

